# Rainfall and habitat interact to affect the condition of a wintering migratory songbird in The Bahamas

**DOI:** 10.1002/ece3.5359

**Published:** 2019-06-27

**Authors:** Michael E. Akresh, David I. King, Peter P. Marra

**Affiliations:** ^1^ Department of Environmental Conservation University of Massachusetts Amherst Amherst Massachusetts; ^2^ Department of Environmental Studies Antioch University New England Keene New Hampshire; ^3^ U.S. Forest Service Northern Research Station University of Massachusetts Amherst Amherst Massachusetts; ^4^ Migratory Bird Center Smithsonian Conservation Biology Institute Washington District of Columbia

**Keywords:** climate change, nonbreeding, Prairie Warbler, precipitation, sex ratios, stable isotopes

## Abstract

On the subtropical and tropical wintering grounds of migratory birds, variation in moisture levels and habitat can influence the availability of food resources and subsequently impact overwintering birds. Using stable carbon isotopes in blood samples as a measure of moisture, we assessed the interactive effects of rainfall, vegetation, and moisture on the demographics and condition of Prairie Warblers (*Setophaga discolor*) wintering in The Bahamas. Carbon isotopes in Prairie Warbler blood were more depleted in taller, wetter habitats; we additionally detected novel temporal effects of rainfall on isotope values. During a winter with more rainfall, most birds maintained mass and pectoral muscle regardless of the habitat type occupied. In a winter with less rainfall, birds lost mass and pectoral muscle, and this effect was more pronounced in birds with enriched isotope values and birds that occupied drier, shorter habitat. Prairie Warblers exhibited strong patterns of sexual habitat segregation with males disproportionately observed in areas with taller vegetation and females in shorter vegetation. During the drier winter, older males had better maintenance of pectoral muscle compared to females and younger individuals. Also in the drier winter, daily rainfall patterns explained more of the variation in body condition compared to the date of capture; pectoral muscle was best explained by recent precipitation (during the previous 30 days), while size‐corrected mass was more a function of longer‐term (90‐day) rainfall and habitat moisture. Our findings along with other studies suggest that Prairie Warblers and other migratory birds are sensitive to interactions between annual variation in winter rainfall, within‐season daily rainfall patterns, and habitat quality. Increasing drought and habitat loss in the Caribbean may be having a negative impact on wintering bird populations. To best conserve Nearctic–Neotropical migratory passerines in the region, we recommend prioritizing the protection of the least drought‐prone wintering areas.

## 1 INTRODUCTION

Migratory birds are vulnerable to changes in both biotic and abiotic factors that influence the quality of their habitats throughout their full annual cycle (Faaborg et al., 2010). For example, wintering migratory passerines can be negatively influenced by the loss and degradation of high‐quality, tropical forests (Rappole & McDonald, 1994), as well as drought (Achard et al., 2014; Studds & Marra, 2011). Gaining a better understanding of how biotic and abiotic factors interact to affect migratory birds throughout their life cycle, including during the less frequently studied wintering season, is increasingly recognized as being important for the conservation of declining populations (Faaborg et al., 2010; Marra, Cohen, Loss, Rutter, & Tonra, 2015a).

A substantial proportion of Nearctic–Neotropical migratory bird species winter in the Caribbean during the tropical dry season (Terborgh, 1989; Wunderle & Waide, 1993). The dry season in most of the Caribbean often begins in November or December and extends to April, although there is some variation among regions and years (Latta & Faaborg, 2001; Sealey, 2006; Studds & Marra, 2007). At the end of the dry season (March–April), ecological systems can become stressed due to the lack of moisture (Johnson & Sherry, 2001; Rapport, Regier, & Hutchinson, 1985; Wunderle, Lebow, White, Currie, & Ewert, 2014). Moisture can vary temporally and spatially as a function of rainfall and temperature, as well as due to spatial variation in edaphic conditions and the ability of vegetation to retain moisture (Murphy & Lugo, 1986; Wang, Hall, Cornell, & Hall, 2002). Interactions between spatial and temporal variation in moisture can impact avian food resources, such as insect or fruit (Brown & Sherry, 2006; Strong & Sherry, 2000; Studds & Marra, 2007). For instance, if rainfall amounts decline equally throughout a given area as the dry season progresses, habitats that better retain moisture tend to have more insects and fruit (Parrish & Sherry, 1994; Wunderle et al., 2014). However, annual variation in winter rainfall can also be important for avian food resources and may override other factors. Some years have relatively high amounts of rainfall throughout the dry season, and in these years, food resources may be abundant regardless of the habitat type or the specific time within the dry season (Brown & Sherry, 2006; Studds & Marra, 2007; Wunderle et al., 2014).

Drier conditions resulting from low rainfall, or due to the moisture‐retention capacity of a particular habitat, can negatively affect avian body condition during the wintering period (e.g., Latta & Faaborg, 2001, 2002, Smith, Reitsma, & Marra, 2010, Angelier, Tonra, Holberton, & Marra, 2011). Birds that are unable to maintain their body condition during the winter can have difficulty in building the fat and muscle reserves necessary for migration, and subsequently may depart later for spring migration (Cooper, Sherry, & Marra, 2015; Studds & Marra, 2007, 2011). Moreover, birds that are exposed to drier conditions and lower food availability may have lower survival during a subsequent spring migration (Johnson, Sherry, Holmes, & Marra, 2006; Latta et al., 2016; Studds & Marra, 2005) or have reduced reproductive output on the breeding grounds (Drake, Rock, Quinlan, & Green, 2013; Norris, Marra, Kyser, Sherry, & Ratcliff, 2004; Reudink et al., 2009).

Many migratory bird species segregate by age or sex in wintering habitats that differ in vegetation type, structural characteristics, or moisture (Murphy et al., 2001; Ornat & Greenberg, 1990; Wunderle, 1995). Males and older individuals often occupy taller, wetter vegetation, while females and younger individuals are segregated to poorer‐quality, drier habitats (Marra, 2000; Marra, Sherry, & Holmes, 1993; Mettke‐Hofmann et al., 2015). Habitat segregation is important to consider because it can influence variation among age and sex classes in their ability to maintain body condition during the dry season (Marra & Holmes, 2001), and subsequently their capacity to survive or reproduce successfully (Marra, Hobson, & Holmes, 1998; Norris et al., 2004; Rockwell et al., 2017).

Stable carbon isotope ratios in bird tissues can be used as a proxy for moisture levels within an individual's home range (Smith et al., 2010). Carbon isotope ratios, which can be depicted as δ^13^C, are ratios of the ^13^C and ^12^C molecules within samples (Farquhar, Ehleringer, & Hubick, 1989). Due to different photosynthetic pathways, δ^13^C values in C_4_ and CAM plants, which typically grow in drier habitats, differ compared to δ^13^C values in C_3_ plants, which typically grow in wetter habitats (Dawson, Mambelli, Plamboeck, Templer, & Tu, 2002; Farquhar et al., 1989; Marshall, Brookes, & Lajtha, 2007). Genetic differences among plants growing in wet versus dry habitats can also affect δ^13^C values (Dawson et al., 2002; Johnsen, Flanagan, Huber, & Major, 1999; Zhang, Marshall, & Jaquish, 1993). Lastly, individual plants that are water stressed uptake ^13^C at a different rate during photosynthesis, and as a result, δ^13^C values in plants can be influenced by temporal variation in rainfall (Korol, Kirschbaum, Farquhar, & Jeffreys, 1999; McNulty & Swank, 1995). Because of the above processes, plants in drier habitats, or those experiencing drought conditions, tend to have enriched stable isotope ratios of carbon (δ^13^C; Flanagan & Johnsen, 1995, Stewart, Turnbull, Schmidt, & Erskine, 1995, Korol et al., 1999). In the Caribbean, Marra et al. (1998) observed that δ^13^C values in insects sampled in wet versus dry habitats also matched the gradient of δ^13^C values found in plants. Since wintering migratory passerines are often insectivorous and maintain home ranges, a number of studies have subsequently found that δ^13^C values in tissues of birds sampled along a moisture gradient match the same gradient of δ^13^C observed in plants and insects (Bearhop, Hilton, Votier, & Waldron, 2004; Drake et al., 2013; Marra et al., 1998; Smith et al., 2010; Studds & Marra, 2005). δ^13^C values in bird tissue can therefore be used as an objective measure of the moisture occurring within a bird's home range and can be an especially useful proxy when studying large numbers of inconspicuous, difficult‐to‐track birds.

Given the importance of the wintering period to migratory birds, we examined the critical question of how habitat quality and rainfall, and particularly interactions between these factors, influence overwintering migrants. Specifically, using carbon isotope ratios in bird tissues as a proxy for moisture levels, we assessed the interactive effects of rainfall, moisture, and habitat on the demographics and body condition of a Nearctic–Neotropical, migratory, insectivorous bird, the Prairie Warbler (*Setophaga discolor*), in The Bahamas. Prairie Warblers are well suited for this study because they use a variety of habitat types along a moisture gradient on their wintering grounds (Murphy et al., 2001; Wunderle & Waide, 1993) and can be readily captured, sexed, and aged in the field (Pyle, 1997). Previous studies in The Bahamas and the Dominican Republic observed Prairie Warbler males are more likely to be captured in taller vegetation compared to females (Latta & Faaborg, 2001; Murphy et al., 2001). Additionally in the Dominican Republic, Latta and Faaborg (2001) found that Prairie Warblers maintained pectoral muscle over the winter in high elevation pine forests that received relatively high rainfall amounts, but birds in low elevation, dry desert sites lost muscle over the winter.

**Figure 1:**
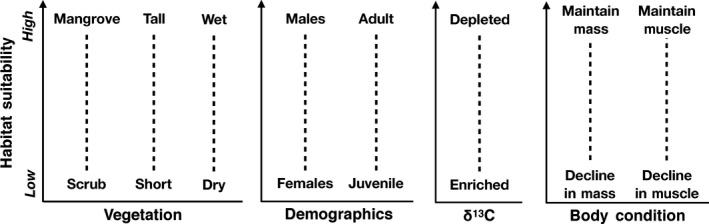
Predicted relationships between Prairie Warbler habitat suitability and vegetation type, height, and moisture levels, underlying demographic population structure, δ^13^C values in blood, and change in overwinter body condition (hypotheses 1–3)

In our study, we assessed a number of a priori hypotheses and predicted outcomes. We hypothesized that (1) Prairie Warblers exhibit habitat segregation during the winter as found previously, and predicted habitats with taller, wetter vegetation (e.g., mangroves and tall coppice) would be occupied by dominant males and older individuals. We proposed that (2a) δ^13^C values in bird tissues would reflect spatial variation in moisture, and expected that tissue samples from birds in taller, wetter habitats would have more depleted δ^13^C values. We also hypothesized that (2b) δ^13^C values would be influenced by temporal variation in moisture, and we predicted more depleted δ^13^C values in tissues sampled earlier in the winter dry season, during wetter years, or during periods of more daily rainfall. We hypothesized that (3) winter habitat interacts with ordinal date to influence body condition, and expected birds with depleted δ^13^C values, or birds that occupied mangroves and taller, wetter vegetation, would maintain mass and muscle throughout the winter, while birds with enriched δ^13^C values, or birds in scrubbier, shorter, and drier habitats, would decline in body condition over the winter dry season (Figure 1). If males and older individuals occupied wetter, taller habitats (hypothesis 1), we expected older males would maintain body condition more so than females and younger birds. In addition to our 3rd hypothesis, we hypothesized that (4) within‐season body condition is better explained by variables relating to daily rainfall patterns compared to a variable of ordinal date. Lastly, previous studies have found support for a final hypothesis that (5) yearly variation in rainfall can override other relationships with body condition (Angelier et al., 2011; Studds & Marra, 2007). We therefore considered that overwintering body condition could be affected by habitat, the specific time during the dry season, and/or daily rainfall patterns only in drier winters, while in wetter winters, all birds maintain condition regardless of any of the other factors (i.e., hypotheses 3 and/or 4 are predicted to be true in dry years, but false in wet years).

Our study complements existing studies on wintering Prairie Warblers (e.g., Baltz, 2000, Latta & Faaborg, 2001) by assessing habitat segregation and overwinter body condition in different habitat types in The Bahamas. Unlike previous studies, we explore novel uses of carbon isotopes and daily rainfall measurements to test how interactions between spatial and temporal variation in moisture affect both pectoral muscle and size‐corrected body mass. Few other studies have assessed the reliability of using δ^13^C values in bird tissue as a proxy for both spatial and temporal variation in moisture on the wintering grounds. Furthermore, few studies on wintering migratory passerines have quantitatively examined within‐season daily rainfall patterns with respect to individual capture dates. Findings from our study should allow researchers and conservationists to better understand the value of various Bahamian habitat types for wintering migratory birds. Considering recent declines in precipitation in the Caribbean (Martin & Weech, 2001; Studds & Marra, 2011) as well as anticipated future declines in rainfall (Neelin, Munnich, Su, Meyerson, & Holloway, 2006), understanding the influence of habitat and moisture on wintering migrants in The Bahamas could be key to their conservation.

## 2 MATERIAL AND METHODS

### 2.1 Study site

**Figure A1:**
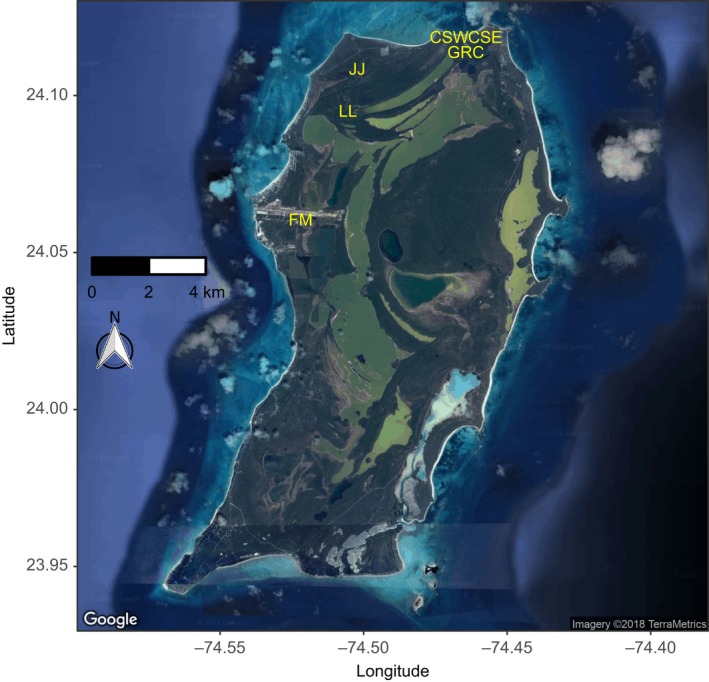
Map of San Salvador Island, The Bahamas, with locations of the six Prairie Warbler study plots (CSW, CSE, GRC, JJ, LL, and FM). The CSW, CSE, and GRC plots were directly adjacent to each other. The map was obtained from Google Earth imagery, using the “ggmap” package in R (Kahle & Wickham, 2013)

Between 2012 and 2015, we conducted our research on 6 study plots in the central to northern part of San Salvador Island (24.12°N, 74.46°W; Figure A1 in Appendix), a low elevation (<50 m asl), relatively small (163 km^2^), outer island in the eastern part of The Bahamas. Plots were selected to encompass a moisture gradient and be representative of the main habitat types found throughout San Salvador and the southern Bahamas (Correll, 1979; Currie et al., 2005; Smith, 1986). These included 2 plots (named CSE, 8 ha, and CSW, 5 ha) in coastal scrub/coppice habitat (Correll, 1979; Smith, 1986) on sandy soils (Mooney, 1905) with low‐growing, native plant species (e.g., *Croton linearis*, *Erithalis fruticosa*,* Pithecellobium keyense*, *Bourreria succulenta*, *Lantana involucrata*). A third site (GRC, 3 ha) was in highly disturbed habitat with many non‐native plants (e.g., *Leucaena leucocephala*, *Casuarina equisetifolia*, *Corchorus hirsutus*, and *Pluchea odorata*). A fourth and fifth site (JJ and LL, both 7 ha) were on black soils consisting of taller native, inland coppice interspersed with some mangroves (e.g., *Rhizophora mangle*), tall buttonwood (*Conocarpus erectus*), and small patches of short coppice. In JJ, there were additionally extensive areas of sabal palm (*Sabal palmetto*) and cocoplum (*Chrysobalanus icaco*) vegetation. Finally, a sixth study site (FM, 7 ha) was located on rocky and clay‐like soils dominated by short buttonwood (1–4 m in height) and sawgrass (*Cladium jamaicense*), but also had patches of non‐native vegetation, short inland coppice, and coastal scrub vegetation, interspersed with small ponds (Jones, Akresh, & King, 2013).

Daily rainfall data were available from July 2012 to April 2014 from a weather station located at the Gerace Research Centre (i.e., at the GRC plot, directly adjacent to plots CSE and CSW) in the northeastern part of the island (data obtained from D. W. Gamble). The two other plots located in the north part of the island (JJ and LL) were within 5 km of the weather station and these plots likely received similar amounts of rainfall during a given time period. The FM plot was 8 km southwest of the weather station and it is possible that rainfall amounts were slightly different at this location (Gamble & Jordan, 2006). However, we suspect that spatial differences in moisture levels among the different plots and habitat types were primarily a result of the underlying soils and the freshwater table, rather than due to spatial variation in rainfall (Sealey, 2006; Wang et al., 2002).

### 2.2 Bird sampling

Birds were captured primarily during 2 periods, mid‐winter (late December to end of January), and in late winter (25 February to 20 March) in each of 3 winters (2011/2012, 2012/2013, and 2013/2014), and again only in mid‐winter in 2014/2015 (Akresh & King, 2015; Jones et al., 2013). We denote the 2011/2012 winter as “2012,” 2012/2013 as “2013,” and 2013/2014 as “2014.” We conducted mist‐netting only on 4 plots (CSE, CSW, FM, and GRC) in the initial 2012 season, on all plots in 2013 and 2014, and conducted only limited netting in 2015. We used several methods to capture birds (Wunderle et al., 2010). Passive netting was conducted with 7–17 nets (6‐ and 12‐m nets) placed 0–50 m apart and operated for 1–2 mornings (sunrise to 12:00 EST). Nets were moved to systematically cover entire plots during a winter period (mid‐ or late winter). Target netting was also conducted with 1–5 12‐m nets using a decoy and playback of either Prairie Warbler songs and/or chip notes, or alarm “chatter” calls of white‐eyed vireos (*Vireo griseus*). Lastly, passive netting was sometimes augmented with playback, by rotating broadcasted playback among nets for 30–60 min (Wunderle et al., 2010). On plots with tall vegetation (JJ and LL), we sometimes stacked 2 nets on top of each other, with netting up to 5 m in height. For every individual capture, we noted if the bird was caught using passive, target, or the combination of passive and target netting.

We banded each Prairie Warbler with a United States Geological Survey aluminum band and a unique combination of 3 plastic color bands. We classified birds by sex and as either juveniles (<1 year old) or adults (>1 year old) using plumage and molt limits (Pyle, 1997). We also measured unflattened wing chord (±1 mm), tail length (±1 mm), tarsus length (±0.1 mm), and mass (±0.01 g, using a digital scale). We scored furcular and abdominal fat levels using a standardized scale of 0–7 (DeSante, Burton, Velez, Froehlich, & Kaschube, 2009) and, in all winters besides 2012, scored pectoral muscle using a standardized scale between 1 and 4, with 1 = muscle concave, 2 = muscle in a “v” shape, 3 = muscle slightly convex, and 4 = muscle highly convex (Cooper et al., 2015; Latta & Faaborg, 2001). We also used mid‐level scores (i.e., 1.5, 2.5, and 3.5) when pectoral muscle was in between the above categories. To reduce interobserver variation, 98% of birds were measured and scored by a single observer (MA).

For a subset of the captured birds, we were successful in collecting blood samples (up to 50 µl) from the brachial vein using capillary tubes. The blood was then put on ice and later transferred to 1.5‐ml microcentrifuge tubes before placing in a freezer. In 2012 and 2013 for a subset of birds (birds captured in different habitat types throughout the winter season), we also sampled the tip (2–3 mm) of the central claw on both feet of the individual. We sampled both blood and claws because carbon isotope ratios in whole blood (plasma and red blood cells combined) have a short half‐life (i.e., 4–11 days; Pearson, Levey, Greenberg, & Rio, 2003, Evans Ogden, Hobson, & Lank, 2004) and whole blood likely represents dietary isotopic input from a few weeks before capture to the time of capture, whereas isotope ratios in claw tips have a relatively long half‐life (i.e., 27 days; Lourenço, Granadeiro, Guilherme, & Catry, 2015) and the isotope ratios likely represent diet a few weeks to a few months before capture (Bearhop, Furness, Hilton, Votier, & Waldron, 2003; Hahn, Dimitrov, Rehse, Yohannes, & Jenni, 2014).

In late January and February 2012–2014 and in January 2015, we conducted intensive area searches within the plots (Latta & Faaborg, 2001; Marra, 2000) where we resighted color‐banded Prairie Warblers captured in mid‐winter or in previous years. We followed similar methods as previous studies, searching along a grid of trails in each plot and adjacent areas beginning at sunrise until bird activity declined (around 10:00 EST), and then again in the late afternoon when bird activity picked up (approximately 15:30 to sunset). Plots were usually surveyed for five days during a given winter, in which the plot was surveyed for approximately 10 person‐hours per day. Averaging across all years and plots, we surveyed individual plots for 51.7 (*SD* = 12.5) person‐hours per winter. At each Prairie Warbler sighting, observers recorded the color band combination. Observers also recorded the foraging substrate and estimated the height of the bird (often 5–10 s after recording the color bands), in order to determine whether different age and sex classes were observed at different heights in the vegetation. Most birds were observed briefly (78% were tracked for a minute or less), and only one observed height for each sighting was used in our analyses. We suspect that the recorded height of the bird was generally not biased due to detectability and was not influenced by our initial presence. For a subset of sightings, we recorded multiple heights of the bird by following the same individual for 1 to 15 min; 77% of the subsequent recorded height observations (*n* = 47) were within 1 m of the original height recorded for that sighting.

This work was executed in accordance with and approval of the proper animal care and use protocols and national/international permits, including the University of Massachusetts Amherst Institutional Animal Care and Use Committee and the U.S. Federal Bird Banding Program.

### 2.3 Vegetation sampling

At each net lane within plots, we estimated the average height of the canopy with a 3 m pole and recorded 3–4 of the dominant plant species within 2 m of the net lane. Vegetation at each net lane was later classified as one of the following habitat types based on the dominant species and descriptions of vegetation communities in the literature (Correll, 1979; Currie et al., 2005; Smith, 1986): coastal scrub (otherwise known as coastal coppice), disturbed/non‐native vegetation, short coppice (defined as inland coppice <3 m in height), tall coppice (inland coppice >3 m in height), short buttonwood/sawgrass‐dominated vegetation, and mangrove/sabal palm/tall buttonwood/cocoplum‐dominated vegetation. Net lanes were distributed throughout the plots and were representative of the spatial variation of the vegetation within each plot. To compare whether the vegetation at capture locations was similar to the vegetation used by the birds in the rest of their home ranges, we also classified the foraging substrate of resighted birds into these same habitat types.

### 2.4 Stable isotope preparation and analysis

Claws were soaked for 2 hr in a 2:1 chloroform:methanol solution and dried in a fume hood for 48 hr. Blood was freeze‐dried while in microcentrifuge tubes and then powdered. All samples were then weighed in tin capsules and combusted in a continuous flow isotope ratio mass spectrometer (Thermo Scientific Delta V Advantage mass spectrometer coupled with a Costech ECS 4010 elemental analyzer via a Conflo IV gas interface) at the Stable Isotope Mass Spectrometry Facility at the Smithsonian Institution, Suitland, MD, USA. One in‐house standard was run for every 4 unknowns. Stable isotope ratios (^13^C/^12^C) are reported in delta (δ) notation, in per‐mil units (‰) relative to the Vienna Pee Dee Belemnite (δ^13^C) standard. Repeated analyses of blood samples from the same individual capture were replicable to within 0.2‰ (*n* = 25).

### 2.5 Statistical analysis

#### 2.5.1 Age/sex ratios

For the following analyses, we compared differences in sex (male vs. female), age (juvenile vs. adult), and age/sex (four separate groups). We tested whether age/sex ratios of captured Prairie Warblers varied with the vegetation type at the capture location using chi‐squared tests or varied with vegetation height at the capture location using mixed models with individual as a random effect. We also examined whether δ^13^C values of blood (as an index of habitat wetness) differed among age/sex classes using mixed models, including covariates of ordinal date and year to account for temporal variation in δ^13^C values, and also including individual as a random effect. A previous study on Kirtland's Warblers (*Setophaga kirtlandii* ) observed that the use of playback may bias captures toward males (Wunderle et al., 2010), but we found Prairie Warbler age/sex ratios were not affected by the use of playback during capture and age/sex ratios also did not differ in plots among years (chi‐squared tests within individual plots, all *p* > 0.05). Thus, we compared the differences in age/sex ratios with birds captured using all netting methods and in all years combined. We did not include within‐year recaptures in the above analyses, but the same individual captured in different years (6% of the population) was considered a unique sample each year because a bird's age class was often dependent on the capture year. Moreover, we were able to include a random effect of individual in most models, which accounted for any pseudo‐replication of sampling the same individual over multiple years.

We also tested whether different age/sex classes were observed at different heights in the vegetation with the observational data of color‐banded birds. We conducted mixed models with height of the bird as the response variable, sex, age, or age/sex as the predictor variable, and a random effect of individual. As in the other models, the random effect of individual accounted for the nonindependence of multiple observations of the same individual within or among years. Both vegetation height and age/sex ratios varied among plots, and we therefore did not include a correlated variable of plot in the models because we were primarily interested in examining variation in bird occupancy among vegetation heights. For the mixed models using captures or resighting data, we used likelihood ratio tests to test for significant differences among the 4 separate age/sex groups, by comparing a model with and without a covariate of age/sex groups. We used the R statistical program version 3.3.1 to conduct all analyses (R Core Team, 2016) and used the “lme4” and “lmerTest” packages (Bates, Maechler, Bolker, & Walker, 2015; Kuznetsova, Brockhoff, & Christensen, 2016) to conduct mixed models. Results are presented as means ± SE. We defined significant results as *p* < 0.05.

#### 2.5.2 δ^13^C values

We tested whether δ^13^C values from captured Prairie Warblers (δ^13^C values are from blood unless otherwise noted) were a function of spatial variation in moisture, as reflected by vegetation type or vegetation height, and temporal variation in moisture, as reflected by capture date, rainfall, or year. We used linear mixed models with δ^13^C values as the response variable and, in separate models, examined the capture net vegetation type or height as the main predictor variable, while also taking into account temporal effects of year and capture date. We also tested the effect of rainfall on δ^13^C values in 2013 and 2014, with a main predictor variable of total rainfall at either 30, 60, 90, or 120 days prior to capture (removing the correlated capture date variable from the models), and included covariates of year and vegetation type (Wunderle et al., 2014). In all models, we included a random effect of individual; we did not include plot in the models because the vegetation at the net lanes was correlated with plot. We used the “multcomp” package (Hothorn, Bretz, & Westfall, 2008) to conduct Tukey's tests to examine pairwise comparisons among years and vegetation types while controlling for the family‐wise error rate.

To further examine temporal changes in δ^13^C values, using paired *t* tests we compared δ^13^C values of blood between mid‐winter and late winter for the same individual birds captured during both periods. Using a linear mixed model with individual as a random effect, we also tested the relationship between δ^13^C values of blood versus claws taken at the same capture from an individual bird. Since claws represent a different dietary time‐span compared to blood (Lourenço et al., 2015), we would interpret a strong correlation as an indication that an individual bird was using habitat of the same amount of wetness throughout the previous few months and that there was little temporal change in δ^13^C values over the previous few months.

#### 2.5.3 Body condition

We computed a body size index using the first principal component (PC1) from a principal component analysis of body size measurements (wing, tail, and tarsus length; PC1 explained 62% of the variance). We then computed a size‐corrected body mass index taking the residuals from the linear relationship of PC1 regressed over mass (*r* = −0.50, *p* < 0.001; Marra et al., 1998, Wunderle et al., 2014). Although we note all size‐corrected mass indexes have some caveats (Labocha, Schutz, & Hayes, 2014; Schamber, Esler, & Flint, 2009), solely using body mass would not account for covariation with size, and we could not conduct a multiple regression approach (Labocha et al., 2014) because we were using our index as a response variable. Moreover, we chose the above residual‐based index over the newer scaled mass index (Peig & Green, 2009) because our approach allowed us to correct for body size using multiple body size metrics (Jacobs et al., 2012), had fewer allometric assumptions (e.g., the assumption of a nonlinear relationship between mass and size did not appear to fit our data), and had an resulting index with a lower correlation with body size (*r* = 0.0 compared to *r* = −0.48 for the scaled mass index using wing chord), and a higher, more biologically intuitive correlation with body mass (*r* = 0.87 compared to *r* = 0.50 for the scaled mass index).

We used linear mixed models to examine change in the size‐corrected mass index and pectoral muscle shape over the winter season in the population, with size‐corrected mass or pectoral muscle score as the response variable, and day of season as the main predictor variable. We further tested the effect of day of season on size‐corrected mass and muscle separately for each age/sex class. In separate analyses, we also tested the effect of an interaction of day of season and either vegetation height, or vegetation type, at the capture net, or δ^13^C values of blood (indicating habitat moisture) on size‐corrected mass or muscle score. Since the vegetation variables and δ^13^C values were partially correlated, we conducted separate models with each individual covariate to prevent multicollinearity. For all models, we analyzed individual years separately because we often observed different results (i.e., interactions) among years. In all models with the size‐corrected mass index as the response variable, we included an additional covariate of time after sunrise because birds caught later in the day were in better condition (*ß* = 1.23 ± 0.24, *t* = 5.1, *p* < 0.001). In almost all models, we included a random effect of individual bird. In the few cases when mixed‐effects models did not converge well (when there were few recaptured individuals), we ran simpler fixed‐effects models without the random effect of individual.

We excluded any recaptures of the same individual captured multiple times on the same day. We also excluded captures in late March (20–25 March), when we only had a few captures and some birds were putting on large fat loads before migration. Due to small sample sizes, we did not include captures in January 2015, or examine the effects of day of season on the size‐corrected mass index or pectoral muscle within the short coppice or mangrove/sabal palm vegetation type in any year, or within the tall coppice in 2012. We could only include birds with blood samples in the analyses with δ^13^C values and only had data to examine muscle in 2013 and 2014. Since 93% of included captures had a fat score of 0 or 1 (indicating a trace or no fat), our fat scoring classification system was likely too coarse (Cooper et al., 2015) and we therefore did not further analyze fat scores.

We used vegetation height and type at the capture location, and δ^13^C values, as proxies for the habitat in an individual's entire home range. We note that for a small subset of individuals, the vegetation at the capture location may not have represented the vegetation used by the bird in its entire home range. Nonetheless, four of our plots were fairly homogeneous in habitat type (Table 1); therefore, the habitat at the capture location was embedded within the same adjacent habitat type. Furthermore, color‐banded birds captured in mid‐winter were often resighted in the same vegetation type as recorded at the capture location. Specifically, for individuals that were originally captured mid‐winter in coastal scrub and then resighted, 91% of these individuals were primarily resighted in this same habitat type. Similar percentages were found for birds captured and resighted in mangroves and sabal palms (80%), in short buttonwood vegetation (74%), non‐native vegetation (69%), and in tall coppice (55%), but not in short coppice (0%). We therefore believe that using the vegetation at the capture net location as a proxy for the habitat in the rest of the bird's home range is warranted, with the exception of short coppice (which had few captures and was excluded when analyzing the effect of vegetation type on body condition). Moreover, the use of proxies allowed us to classify the estimated habitat use of individuals that were not resighted, including birds that were only captured in late winter after our resighting efforts. Besides the vegetation type and height at the capture locations, δ^13^C values provided another measure to explore the effects of habitat and moisture on bird condition.

**Table 1:** Vegetation characteristics at 292 net lanes distributed among the six study plots (CSE, CSW, FM, GRC, JJ, and LL) on San Salvador Island, The Bahamas. Presented are the mean of the canopy height (with SE in parentheses), the *SD* of the canopy height, and the percentage of net lanes in different habitat types within each plot

	CSE	CSW	FM	GRC	JJ	LL
Mean Canopy Height (m)	1.9 (0.1)	2.7 (0.2)	2.7 (0.1)	3.3 (0.2)	4.8 (0.1)	5.1 (0.2)
*SD* Canopy Height (m)	0.5	1.2	0.9	1.4	0.9	1.0
Short Buttonwood (%)	0	0	54	0	3	0
Coastal Scrub (%)	100	84	7	3	0	0
Non‐native (%)	0	2	19	97	0	3
Short Coppice (%)	0	0	20	0	1	0
Tall Coppice (%)	0	14	0	0	43	91
Mangrove and Sabal Palm (%)	0	0	0	0	53	6

In addition to the above body condition analyses, we also wanted to directly assess how daily rainfall patterns affected body condition. Based on previous studies, we hypothesized that total rainfall during the 30, 60, 90, or 120 days prior to an individual's capture date might affect body condition (Angelier et al., 2011; Studds & Marra, 2011). However, many small precipitation events can have the same cumulative rainfall total compared with 1 or 2 large precipitation events, and we hypothesize that the size of precipitation events could influence soil moisture and food resources, and subsequently, body condition (Wunderle et al., 2014). We therefore additionally calculated the standard deviation (*SD*) and the coefficient of variation (CV) of daily rainfall during the 30, 60, 90, or 120 days prior to the capture date. Importantly, *SD* incorporates both the mean and the variation of daily rainfall, while the CV only addresses the variation, independent of the mean, of daily rainfall during a given period. In our study system, large precipitation events are sporadic during the winter; variation in rainfall can effectively quantify the size of precipitation events over a given period because many small precipitation events will have lower variation (i.e., *SD* and CV) compared to one large precipitation event.

Using an information‐theoretic framework, we compared sets of a priori linear mixed models assessing the effect of each of the 12 rainfall variables (total, *SD*, or CV of daily rainfall 30, 60, 90, or 120 days prior to capture) on the size‐corrected mass index or pectoral muscle score. Additionally, we compared models that included rainfall variables and their interactions with habitat wetness (δ^13^C values), vegetation type, or vegetation height at the capture net, because rainfall can interact with habitat to influence body condition (Studds & Marra, 2007). We used Akaike's information criterion corrected for small sample sizes (AIC_c_) and Akaike's model weights (ω*_i_*) to compare among the models and determine whether a certain habitat variable interacted with rainfall to best explain the variation in mass or muscle (Burnham & Anderson, 2002). In our model set, we included additional models with a day of season variable (instead of a given rainfall variable) because we wanted to determine whether the rainfall variables outperformed day of season in predicting size‐corrected mass or muscle. We included an additional covariate of time after sunrise in all the size‐corrected mass models, and we included a random effect of individual bird in all models. We tested for multicollinearity within models; correlation between predictor variables within the separate models was relatively low (*r* < 0.4). We did not include predictor variables that were moderately or highly correlated in the same model (e.g., rainfall variables and day of season). Including the null model, our total model set for a given year and condition index consisted of 53 models (Table A1 in Appendix). We analyzed 4 different sets of these 53 models: Size‐corrected mass index and muscle score were analyzed separately, and we tested the 2 years with rainfall data (2013 and 2014) in separate model sets because we observed different results between years and we were primarily interested in within‐season effects. We note our model sets were extensive; however, few previous studies have examined the effects of rainfall on bird condition with a quantitative approach, and we thus could not refer to the literature to establish a smaller set of a priori models. To maintain consistent datasets to compare models with AIC_c_, we only included captures with δ^13^C values (those with blood samples) in our models. To assist with model convergence, we standardized all continuous predictor variables to a mean of 0 and *SD* of 1 (Bates et al., 2015). We presented models that were less than 2 AIC_c_ from the null model and within 2 AIC_c_ from the best model (Arnold, 2010), for a given model set.

We likely captured a mixture of site‐faithful and “floater” individuals throughout the winter (Brown & Long, 2007; Latta & Faaborg, 2001), but in the above analyses, we did not differentiate between site‐faithful and “floater” birds. We documented site‐faithful, likely territorial, birds that were captured in December or January and resighted or recaptured in February or March on the same plot (59% of mid‐winter captures). We additionally captured individuals in mid‐winter that were not detected later in the season (41%), but we suspect that many of these birds were indeed territorial and we were just unable to detect them after capture. Prairie warblers can be very inconspicuousness on their wintering grounds (Staicer, 1992), especially in the dense vegetation in our study plots. Although three of the plots were directly adjacent to each other (GRC, CSE, and CSW), there was only a single occurrence in which a bird was captured in mid‐winter in one plot and then resighted/recaptured in a different plot (<1% of resighted/recaptured individuals). For Hermit Thrush (*Catharus guttatus*) and Ovenbirds (*Seiurus aurocapilla*), Brown and Long (2007) observed that mark–recapture/resight methods overestimated the frequency of floaters in wintering populations by 200%–400%. Thus, the percentage of floaters in our population was likely well below 41%. Detectability in resighting and recapturing birds in February and March varied among plots and years in our study, confounding any simple estimates of the actual proportion of floaters. Moreover, a number of mid‐winter captures were resighted in February, but were not recaptured in March, and we are unsure if these birds dispersed off plots in March or were just adept at avoiding the mist‐nets in late winter. Lastly, we could not classify many unbanded birds captured in March—these birds were either nonterritorial and not present within plots in mid‐winter, or were site‐faithful, but we failed to capture them in mid‐winter (Brown & Long, 2007). We therefore chose to combine all individuals when analyzing body condition in the entire population, and separately analyzed the site‐faithful, recaptured birds (see below; McKinnon, Rotenberg, & Stutchbury, 2015).

We examined changes in individuals that were captured in mid‐winter and then recaptured in late winter during the same year. We calculated the change in mass (late winter mass minus mid‐winter mass) and the change in muscle score for each individual bird. We then divided the change in mass or pectoral muscle by the number of days between the 2 captures, to account for capture dates and obtain daily rates of change in mass and pectoral muscle for each individual (McKinnon et al., 2015). We first examined annual variation in the rate of change in mass and muscle. In separate models, we then examined the effect of vegetation height or δ^13^C value on the rates of change in mass, taking into account year as a covariate. We ran similar models examining effects on the change in muscle, but did not include year as a covariate because the effect of year was not significant. Given variation in rainfall among years, we also subset the data and examined separate models for each year. All individuals were captured and recaptured in a given winter only once during the study; thus, we conducted linear models without a random effect of individual. For models with δ^13^C values, we used the δ^13^C values from blood samples of mid‐winter captures (Smith et al., 2010); captures without mid‐winter blood samples were omitted. We did not test the effects of vegetation type and age/sex class due to small sample sizes.

## 3 RESULTS

### 3.1 Rainfall

**Figure 2:**
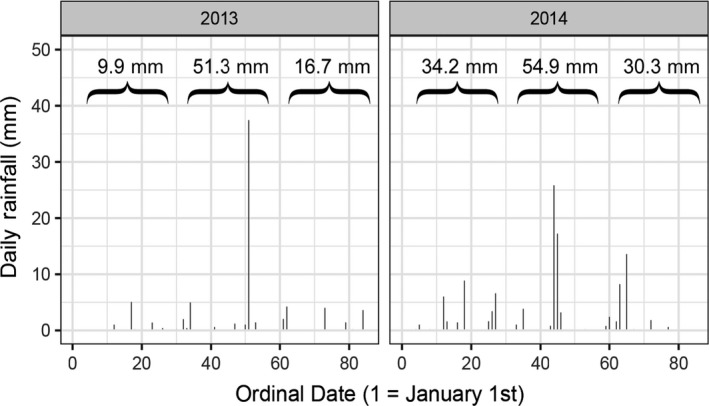
Daily rainfall (mm) between January and March, in the 2012–2013 and 2013–2014 winters. Header values are total monthly values, but for March, the summed values only include rainfall up until 25 March because we did not capture birds past this date

Monthly rainfall totals were higher in the fall and early winter of the 2012–13 season (September = 146.3 mm, October = 358.0 mm, November = 127.3 mm, December = 57.8 mm) compared with 2013–14 (September = 41.5 mm, October = 252.6 mm, November = 102.4 mm, December = 15.7 mm), but rainfall totals in mid‐ to late winter (1 January to 25 March) were lower in the 2012–13 season compared to 2013–2014 (Figure 2).

### 3.2 Vegetation

We recorded the vegetation canopy height and habitat type at 292 net lanes in our 6 study plots. The mean and variance of the vegetation canopy height varied among plots (Table 1). Four plots (CSE, CSW, DistGRC, and LL) consisted predominantly of a single habitat type, while the other 2 plots (FM and JJ) had more variation of habitat within the plot (Table 1).

### 3.3 Sex and age habitat segregation

**Figure 3:**
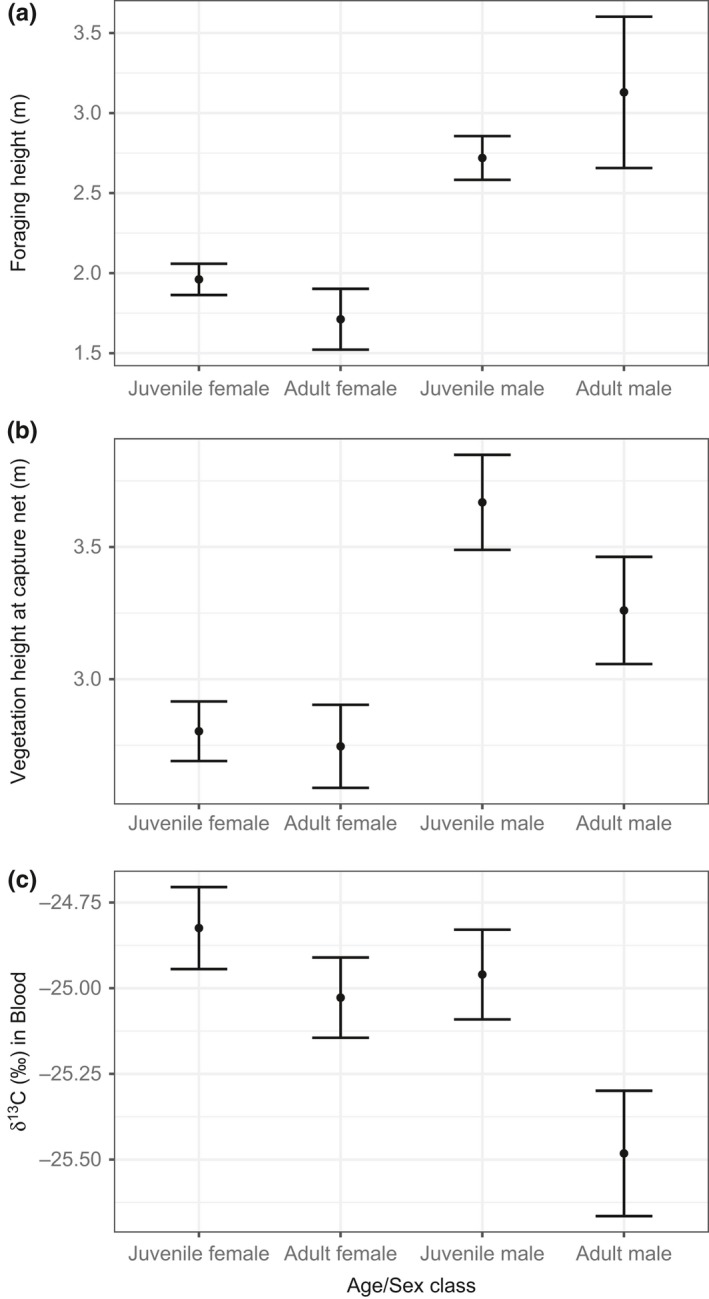
For different Prairie Warbler age and sex classes: (a) Foraging height (m) of resighted, color‐banded birds, (b) vegetation canopy height (m) at capture locations, and (c) δ^13^C values in blood (more negative indicating wetter habitat). Points represent means, and error bars are ± 1 *SE*

We found significant patterns of habitat segregation by sex and age classes. We captured 314 individuals on 334 occasions (excluding within‐year recaptures) in 2012–2015, of which 66% were juveniles and 59% were females. The 4 age/sex classes were captured in net lanes with different vegetation canopy height (*χ^2^* from likelihood ratio test = 24.0, *p* < 0.001; Figure 3); males were captured in significantly taller vegetation (*t* = 4.7, *p* < 0.001), but there was no difference between adults and juveniles (*t* = 0.9, *p* = 0.38). Captures of age/sex classes also differed among nets located in different vegetation types (*χ^2^* = 41.4, *p* < 0.001; Table A2 in Appendix); males were more likely to be captured in mangroves, sabal palms, and taller coppice (*χ^2^* = 28.9, *p* < 0.001), although adults were not captured in different vegetation types compared to juveniles (*χ^2^* ^^= 6.2, *p* = 0.29). δ^13^C values in blood also significantly differed among age and sex classes (*n* = 238, *χ^2^* ^^= 8.0, *p* = 0.05, Figure 3), while accounting for ordinal date and year. There were trends in which males had more depleted δ^13^C values compared to females (*t* = −1.9, *p* = 0.06), indicating males’ home ranges were in wetter habitat, and adults had more depleted δ^13^C values compared to juveniles (*t* = 1.7, *p* = 0.09).

We resighted color‐banded birds and recorded their observed height in the vegetation for 113 individuals on 360 occasions. We found significant differences among age/sex classes (*χ^2^* ^^= 16.0, *p* = 0.001; Figure 3). Males were observed at significantly taller heights compared to females (*t* = 3.8, *p* < 0.001), but there was no difference between adults and juveniles (*t* = −0.4, *p* = 0.66).

### 3.4 Spatial and temporal effects on δ^13^C values.

**Figure 4:**
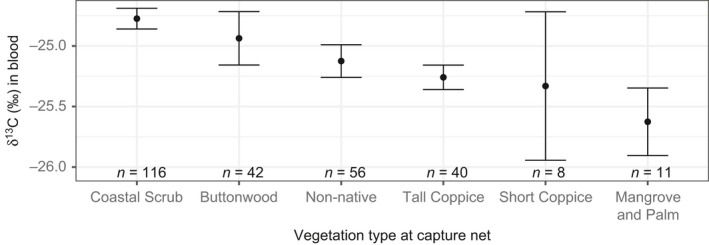
Relationship between δ^13^C values in Prairie Warbler blood with the vegetation type at the capture net in 2012–2015. Points represent means, and error bars are ± 1 *SE*

Prairie Warblers captured in coastal scrub had blood more enriched in δ^13^C (indicating drier home ranges) compared to birds captured in tall coppice (*z* = −4.3, *p* < 0.001), disturbed/non‐native vegetation (*z* = −3.3, *p* = 0.01), and mangrove/sabal palms (*z* =  −2.9, *p* = 0.04), but other pairwise comparisons among vegetation types were not significant (all *p* > 0.05; Figure 4). Birds captured at net lanes with a taller vegetation canopy had depleted δ^13^C values (indicating more moisture; *n* = 273, *ß* = −0.19 ± 0.04, *t* = −4.6, *p* < 0.001).

**Figure 5:**
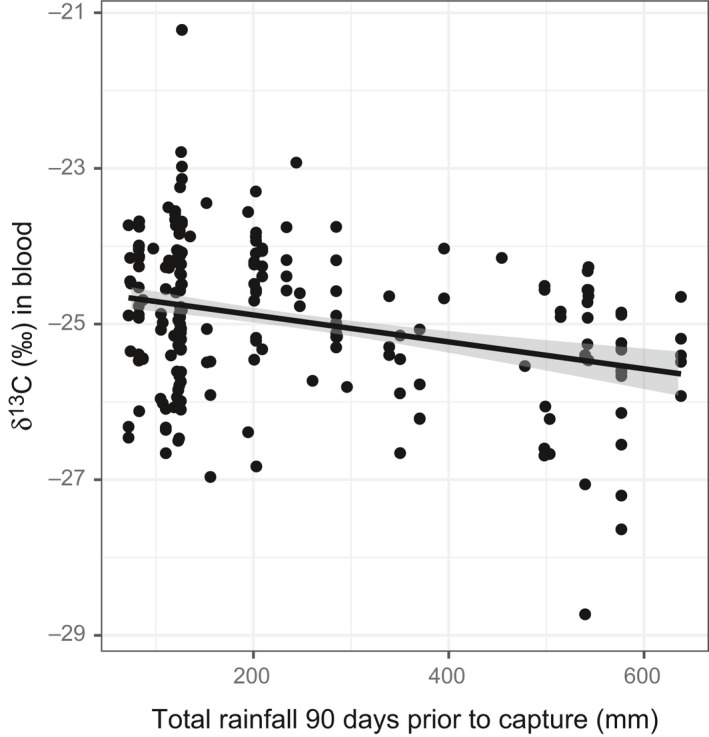
Total rainfall during the 90 days prior to capture and effect on δ^13^C values in Prairie Warbler blood in 2013 and 2014. Solid line represents simple regression line, while gray shading represents the 95% CI

Examining temporal variation, δ^13^C values in blood differed significantly among years with 2014 and 2015 having enriched values (mean in 2014 = −24.65‰ ± 0.09, mean in 2015 = −24.53‰ ± 0.17) compared to 2012 and 2013 (mean in 2012 = −25.29‰ ± 0.17, mean in 2013 = −25.26‰ ± 0.10; the four pairwise comparisons, *p* < 0.001), but there was no difference between 2012 and 2013 or between 2014 and 2015 (*p* > 0.05). δ^13^C values in blood were not significantly affected by rainfall occurring 30 days prior to capture (*n* = 206, *t* = 0.1, *p* = 0.90). However, increased rainfall during 60, 90, and 120 days prior to capture led to more depleted δ^13^C values (60 days: *t* = −2.0, *p* = 0.05, 90 days: *t* = −3.7, *p* < 0.001, Figure 5, 120 days: *t* = −3.4, *p* < 0.001). δ^13^C values at the population level also became more enriched (indicating drying) as the winter progressed (*t* = 4.3, *p* < 0.001). However, for 22 individuals that we captured in mid‐winter and recaptured in late winter, δ^13^C values in mid‐winter (mean = −25.3‰) did not significantly differ from δ^13^C values in late winter (mean = −25.2‰) for the same individual bird (*t* = −0.8, *p* = 0.46), and the correlation between the values was high (*r* = 0.67). We examined δ^13^C values in both claw and blood samples from 67 captures; δ^13^C values in claws highly predicted δ^13^C values in blood (*ß* = 0.95 ± 0.07, *t* = 13.2, *p* < 0.001, *r* = 0.86).

### 3.5 Body condition in the population

**Figure 6:**
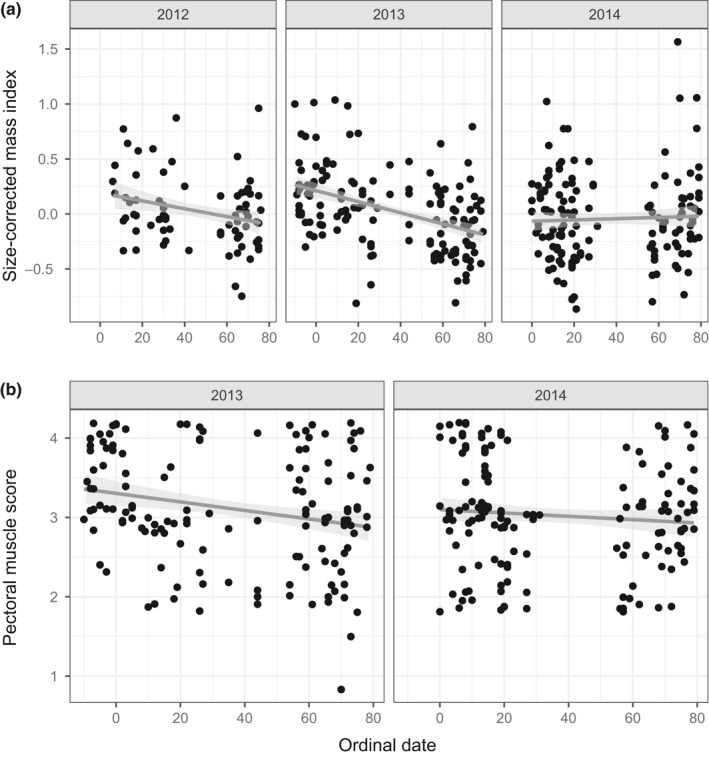
(a) Prairie Warbler size‐corrected mass index as a function of ordinal date in 2012, 2013, and 2014. Solid lines represent simple regression lines, while gray shading represents the 95% CIs. (b) Prairie Warbler pectoral muscle as a function of ordinal date in 2013 and 2014. Points are vertically spread (“jittered”) slightly to facilitate viewing

The body condition of Prairie Warblers changed over the winter season differently depending upon the year. Combining across all individuals, from mid‐ to late winter size‐corrected mass declined in 2013 (*n* = 140, *ß* = −0.0052 ± 0.0007, *t* = −7.2, *p* < 0.001; Figure 6a), nonsignificantly declined in 2012 (*n* = 73, *ß* = −0.0021 ± 0.0012, *t* = −1.8, *p* = 0.08), and nonsignificantly increased in 2014 (*n* = 145, *ß* = 0.0017 ± 0.0010, *t* = 1.7, *p* = 0.10). Similarly, Prairie Warblers lost pectoral muscle over the winter in 2013 (*ß* = −0.0051 ± 0.0018, *t* = −2.7, *p* = 0.007; Figure 6b), but maintained pectoral muscle in 2014 (*t* = −0.7, *p* = 0.51). These patterns in body condition corresponded to variation among years in the amount of rainfall occurring in mid‐ to late winter.

Body condition changed at different rates within age/sex classes (Table 2). In 2012, size‐corrected mass declined over the winter in adult females, while birds of other age/sex classes maintained mass. In 2013, size‐corrected mass declined over the winter for all age/sex classes, and muscle declined for juvenile females, juvenile males, and adult females, but increased for adult males. In 2014, juvenile males increased size‐corrected mass over the winter, while other age/sex classes maintained mass. Juvenile males, adult males, and adult females maintained muscle over the 2014 winter, but muscle declined in juvenile females.

**Table 2:** Results from models examining Prairie Warbler size‐corrected mass and pectoral muscle over individual winters within age/sex classes or within vegetation types

Year	Condition Type	Subset	*n*	*ß*	*SE*	*t*	*P*	Change
2012	Size‐corrected Mass	Adult M	18	−0.001	0.0029	−0.3	0.74	
**2012**	**Size‐corrected Mass**	**Adult F**	**23**	**−0.0039**	**0.0015**	**−2.6**	**0.02**	**−**
2012	Size‐corrected Mass	Juvenile M	9	−0.0098	0.0041	−2.4	0.06	
2012	Size‐corrected Mass	Juvenile F	23	−0.00035	0.00077	−0.5	0.7	
**2013**	**Size‐corrected Mass**	**Adult M**	**24**	**−0.006**	**0.0013**	**−4.6**	**0.02**	**−**
**2013**	**Size‐corrected Mass**	**Adult F**	**31**	**−0.0038**	**0.0013**	**−2.9**	**0.009**	**−**
**2013**	**Size‐corrected Mass**	**Juvenile M**	**35**	**−0.0056**	**0.0018**	**−3**	**0.006**	**−**
**2013**	**Size‐corrected Mass**	**Juvenile F**	**50**	**−0.0065**	**0.0012**	**−5.4**	**<0.001**	**−**
2014	Size‐corrected Mass	Adult M	17	0.0084	0.0039	2.2	0.06	
2014	Size‐corrected Mass	Adult F	16	0.001	0.0033	0.3	0.77	
**2014**	**Size‐corrected Mass**	**Juvenile M**	**51**	**0.0036**	**0.0016**	**2.3**	**0.03**	**+**
2014	Size‐corrected Mass	Juvenile F	61	−0.00079	0.0012	−0.6	0.53	
**2013**	**Pectoral Muscle**	**Adult M**	**24**	**0.0096**	**0.0046**	**2.1**	**0.05**	**+**
**2013**	**Pectoral Muscle**	**Adult F**	**31**	**−0.0083**	**0.0037**	**−2.3**	**0.03**	**−**
**2013**	**Pectoral Muscle**	**Juvenile M**	**35**	**−0.0067**	**0.0031**	**−2.2**	**0.05**	**−**
**2013**	**Pectoral Muscle**	**Juvenile F**	**50**	**−0.0078**	**0.0027**	**−2.9**	**0.007**	**−**
2014	Pectoral Muscle	Adult M	17	−0.0038	0.0047	−0.8	0.44	
2014	Pectoral Muscle	Adult F	16	0.0013	0.0075	0.2	0.87	
2014	Pectoral Muscle	Juvenile M	51	0.0023	0.003	0.8	0.45	
**2014**	**Pectoral Muscle**	**Juvenile F**	**61**	**−0.0069**	**0.0024**	**−2.9**	**0.009**	**−**
2012	Size‐corrected Mass	Buttonwood	15	−0.0056	0.0034	−1.7	0.13	
2012	Size‐corrected Mass	Coastal Scrub	39	−0.0017	0.0015	−1.1	0.29	
2012	Size‐corrected Mass	Non‐native	12	−0.003	0.0045	−0.7	0.52	
**2013**	**Size‐corrected Mass**	**Buttonwood**	**19**	**−0.0098**	**0.0022**	**−4.5**	**0.001**	**−**
**2013**	**Size‐corrected Mass**	**Coastal Scrub**	**57**	**−0.0054**	**0.0009**	**−6**	**<0.001**	**−**
2013	Size‐corrected Mass	Non‐native	29	−0.00016	0.0023	−0.1	0.94	
2013	Size‐corrected Mass	Tall Coppice	21	−0.0014	0.0027	−0.5	0.62	
2014	Size‐corrected Mass	Buttonwood	21	0.0011	0.0018	0.6	0.55	
2014	Size‐corrected Mass	Coastal Scrub	58	0.0023	0.002	1.2	0.25	
2014	Size‐corrected Mass	Non‐native	27	−0.00093	0.0024	−0.4	0.71	
2014	Size‐corrected Mass	Tall Coppice	28	0.0013	0.0023	0.6	0.59	
**2013**	**Pectoral Muscle**	**Buttonwood**	**19**	**−0.013**	**0.0044**	**−2.9**	**0.009**	**−**
**2013**	**Pectoral Muscle**	**Coastal Scrub**	**57**	**−0.0085**	**0.0019**	**−4.6**	**<0.001**	**−**
2013	Pectoral Muscle	Non‐native	29	0.0017	0.006	0.3	0.78	
2013	Pectoral Muscle	Tall Coppice	21	0.0024	0.0088	0.3	0.79	
2014	Pectoral Muscle	Buttonwood	21	0.0022	0.0038	0.6	0.56	
2014	Pectoral Muscle	Coastal Scrub	58	−0.0034	0.0038	−0.9	0.38	
2014	Pectoral Muscle	Non‐native	27	−0.0062	0.0044	−1.4	0.17	
2014	Pectoral Muscle	Tall Coppice	28	0.0035	0.0049	0.7	0.48	

Note

Presented are the data subset, sample sizes (*n*), parameter estimates of the effect of date (*ß*) and their SEs, *t*‐values, and *P*‐values. Models in which we found birds significantly declined (−) or increased (+) condition over the winter are marked in bold and are denoted in the “Change” column

Over the 2012 and 2014 winters, birds captured in all analyzed vegetation types maintained size‐corrected mass and also maintained pectoral muscle in 2014 (Table 2). In 2013, birds captured in tall coppice and non‐native vegetation maintained size‐corrected mass and muscle, but birds in short buttonwood and coastal scrub vegetation declined in mass and muscle over the winter.

**Figure A2:**
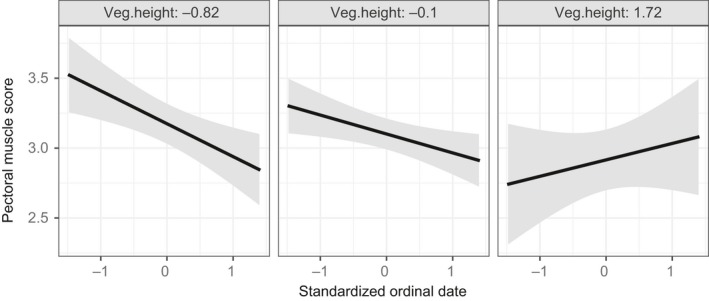
Relationship between Prairie Warbler pectoral muscle score as a function of the interaction between vegetation height at the capture location and ordinal date during the drier winter of 2013. Both predictor variables were standardized with a mean of 0 and a standard deviation of 1. The left facet shows the predicted relationship when the vegetation height is relatively shorter and is set at −0.82 standard deviations below the mean (the 10th quantile). The right facet depicts the relationship in taller vegetation, when the vegetation height is set at 1.72 standard deviations above the mean (the 90th quantile).

There was no significant interaction of vegetation height at the capture location and day of season influencing size‐corrected mass in any year (2012: *t* = 0.3, *p* = 0.77, 2013: *t* = 0.9, *p* = 0.37, 2014: *t* = 1.0, *p* = 0.33). However, in 2013, birds captured in taller vegetation increased muscle over the winter, while birds in shorter vegetation lost muscle over the winter (2013: *t* = 2.0, *p* = 0.04; Figure A2 in Appendix); there was no significant interaction in 2014 (*t* = 1.1, *p* = 0.26).

**Figure 7:**
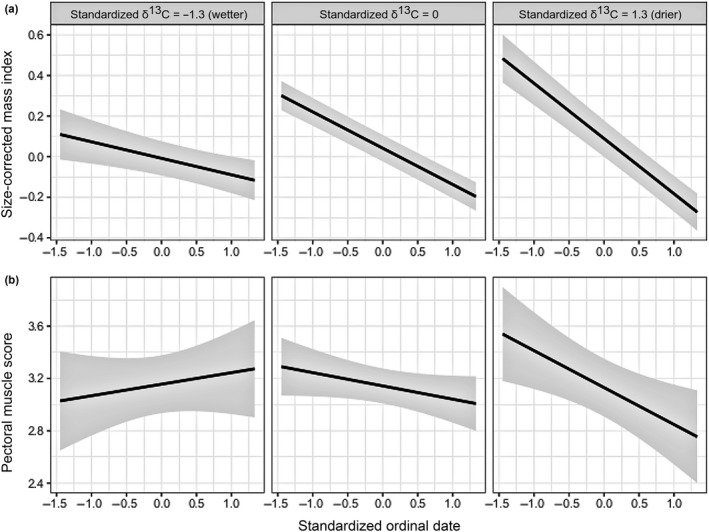
Relationship between (a) the size‐corrected mass index and (b) pectoral muscle as a function of the interaction between δ^13^C values in blood and ordinal date during the drier winter of 2013. Both predictor variables were standardized with a mean of 0 and a standard deviation of 1. The left facets show the predicted relationship between date and condition in wetter habitat, when the δ^13^C values are set at −1.3 standard deviations below the mean (the 10th quantiles). The middle facets depict the relationship when the δ^13^C values set at the mean, while the right facets depict the relationship in drier habitat, when the δ^13^C values are set at 1.3 standard deviations above the mean (the 90th quantiles).

Spatial moisture, as indicated by δ^13^C values in blood, interacted with day of season to influence body mass and muscle, but this relationship varied among years. In the winter of 2013, birds with enriched δ^13^C (indicating drier habitats) had a greater decrease in size‐corrected mass over the winter season compared to birds with depleted δ^13^C (*n* = 99, *t* = −3.1, *p* = 0.02; Figure 7a). Also, birds with enriched δ^13^C (dry habitat) lost muscle over the 2013 winter season, and birds with relatively depleted δ^13^C (wet habitat) gained or maintained muscle (*t* = −2.2, *p* = 0.03; Figure 7b). We found no significant interaction between day of season and δ^13^C values affecting size‐corrected mass in 2012 (*n* = 56, *t* = 0.3, *p* = 0.79) or in 2014 (*n* = 100, *t* = 1.6, *p* = 0.12), or affecting muscle in 2014 (*t* = 1.3, *p* = 0.20).

**Figure A3:**
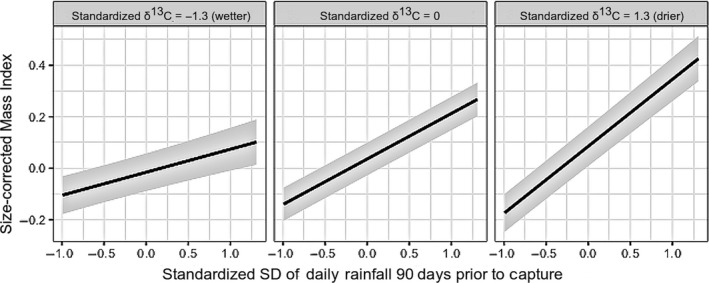
Relationship between Prairie Warbler size‐corrected mass as a function of the interaction between δ^13^C and the *SD* of daily rainfall during the 90 days prior to the capture date in the drier winter of 2013. Both predictor variables were standardized with a mean of 0 and a standard deviation of 1. The left facet shows the predicted relationship between the rainfall variable and size‐corrected mass in wetter habitat, when the δ^13^C value is set at −1.3 standard deviations below the mean (the 10th quantile). The right facet depicts the relationship in drier habitat, when the δ^13^C value is set at 1.3 standard deviations above the mean (the 90th quantile).

**Figure A4:**
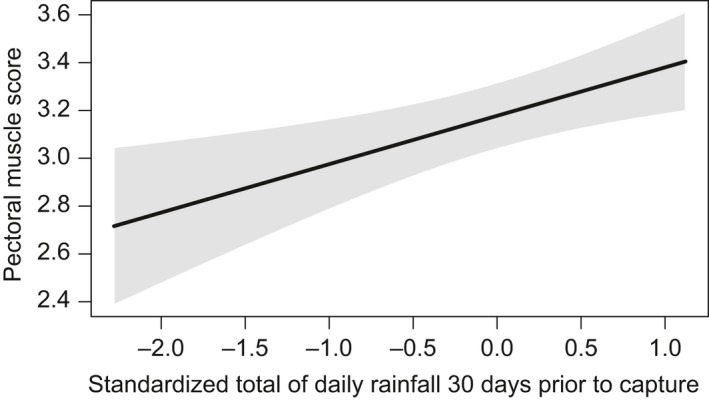
Relationship between Prairie Warbler pectoral muscle as a function of the total daily rainfall during the 30 days prior to the capture date in the drier winter of 2013. The predictor variable was standardized with a mean of 0 and a standard deviation of 1.

Comparing models focusing on the effects of rainfall on size‐corrected mass, in 2013, the model with the lowest AIC_c_ included an interaction between δ^13^C values and the *SD* of the daily rainfall during the 90 days prior to birds’ captures (Table 3); there were no other models within 2 AIC_c_. Birds with enriched δ^13^C values (in drier habitats) declined in size‐corrected mass as the *SD* of daily rainfall decreased, and size‐corrected mass also declined in wetter habitats as a function of the *SD* of rainfall, but at a relatively lower rate of decline (interaction: *t* = 4.9, *p* = 0.009; Figure A3 in Appendix). Examining rainfall effects on pectoral muscle in 2013, the top model had an interaction between vegetation height and the total rainfall during the 30 days prior to birds’ captures (Table 3). Prairie Warblers had larger pectoral muscle when there was more rainfall in the previous 30 days (*t* = 3.2, *p* = 0.002; Figure A4 in Appendix); the interaction with vegetation height was not significant (*t* = 0.2, *p* = 0.85). In 2014 for size‐corrected mass, there were no models less than 2 AIC_c_ compared with the null model, the model without variables besides time since sunrise. In 2014 examining muscle, the best model included total rainfall during the 30 days prior to birds’ captures, with birds surprisingly having larger pectoral muscle when there was less rainfall (*t* = −2.2, *p* = 0.03). In other top models, pectoral muscle decreased as the *SD* of the daily rainfall 30 days prior to birds’ captures increased (*t* = −2.2, *p* = 0.03), and muscle decreased as the CV of the daily rainfall 90 days prior to birds’ captures increased (*t* = −2.1, *p* = 0.04); relationships in the other 3 top models (Table 3) were not significant (all *p* > 0.05).

**Table 3:** The top models (<2 ΔAIC_c_) assessing rainfall variables and date on Prairie Warbler size‐corrected mass and muscle score, separately for each body condition variable, and for 2013 and 2014 (See Table A1 in Appendix)

Subset	Model	*K*	ΔAIC_c_	ω*_i_*
Size‐corrected Mass 2013	90DaySD*δ^13^C^a^	7	0	0.7
Pectoral Muscle 2013	30DayTotal*VegHtatNet^b^	6	0	0.26
	30DayTotal	4	0.22	0.24
Pectoral Muscle 2014	30DayTotal^c^	4	0	0.13
	30DaySD	4	0.27	0.12
	90DayCV	4	0.36	0.11
	90DayCV*δ^13^C	6	0.69	0.09
	90DayTotal	4	1.22	0.07
	90DaySD	4	1.84	0.05

Note

For size‐corrected mass in 2014, no models were < 2 ΔAIC_c _from the null model. Presented are the models, number of parameters (*K*), Akaike's information criterion value corrected for small sample sizes (AIC_c_), difference in AIC_c_ from the top model (ΔAIC_c_), and model weight (ω*_i_*). VegHtatNet = vegetation canopy height at the capture net.

a AIC_c_ = 36.18.

b AIC_c_ = 195.91.

c AIC_c_ = 200.57.

### 3.6 Body condition in individual recaptures

Changes in body mass and pectoral muscle for individuals captured in mid‐winter and recaptured in late winter varied among years: On average, birds maintained body mass over the 2012 winter (*n* = 10, 0.0003 ± 0.0016 g/day), lost mass in 2013 (*n* = 13, −0.0047 ± 0.0011 g/day), and gained mass in 2014 (*n* = 12, 0.0037 ± 0.0019 g/day). Additionally, on average birds in 2013 lost pectoral muscle (−0.0039 ± 0.0048) and in 2014 gained pectoral muscle (0.0023 ± 0.0046).

**Figure 8:**
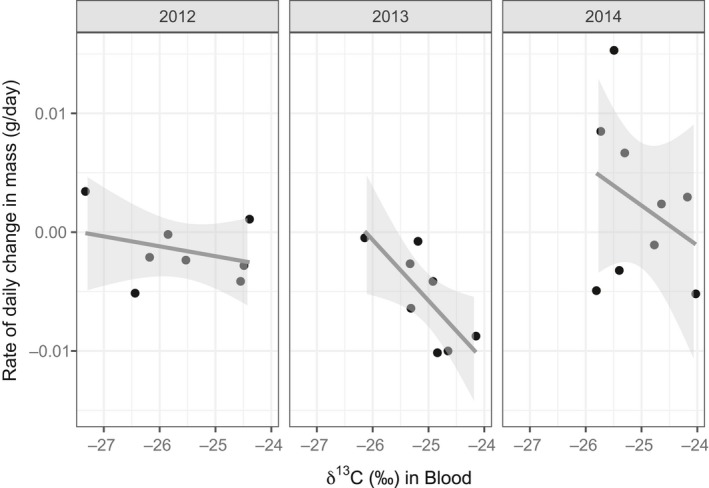
Rate of daily change in mass (g/day) as a function of δ^13^C values in blood, for individuals captured in mid‐winter and recaptured in late winter. Solid lines represent regression lines, while gray shading represents the 95% CI

In all years combined, there were no significant relationships with vegetation height and daily mass change (*t* = 0.0, *p* = 0.99), or daily muscle change (*t* = 1.4, *p* = 0.17), and also no significant relationships were found within years (all *p* > 0.05). Combining across years, there was a trend in which birds with enriched δ^13^C values (in drier habitat) lost on average more mass over the winter, but this was not significant (*n* = 25*, t* = −1.7, *p* = 0.1). There was also a nonsignificant trend in which birds with enriched δ^13^C values lost more muscle, combining across years (*t* = −1.7, *p* = 0.11). Nevertheless, in the drier winter of 2013, birds with enriched δ^13^C values lost significantly more mass (*n* = 8, *ß* = −0.0051 ± 0.0018, *t* = −2.8, *p* = 0.03; Figure 8); there was no relationship in 2012 (*n* = 8, *t* = −0.8, *p* = 0.45) or 2014 (*n* = 9, *t* = −0.9, *p* = 0.40). δ^13^C values were not significantly correlated with daily muscle change in 2013 (*t* = −0.5, *p* = 0.61) or 2014 (*t* = −1.5, *p* = 0.17).

## 4 DISCUSSION

Our final hypothesis (hypothesis 5) was supported by our findings, given that Prairie Warblers maintained body condition in all habitats in a wetter winter (2014), but body condition declined in drier habitats during a drier winter (2013). In The Bahamas, annual variation in winter rainfall is therefore important in influencing the degree to which spatial variation in moisture influences body condition. Our results are consistent with findings of other wintering Nearctic–Neotropical migratory passerines in the Caribbean; similar patterns have been observed for American Redstarts, Ovenbirds, Northern Waterthrushes (*Parkesia noveboracensis*), Cape May Warblers (*Setophaga tigrina*), and Kirtland's Warblers (Brown & Sherry, 2006; Latta & Faaborg, 2001, 2002; Marra & Holmes, 2001; Marra, Studds, et al., 2015b; Smith et al., 2010; Studds & Marra, 2007, 2011; Wunderle et al., 2014). Collectively, these results emphasize that rainfall and habitat moisture underlie the overwinter condition of migratory birds, and the observed patterns are likely due to the influence of moisture on food availability (Brown & Sherry, 2006; Wunderle et al., 2014).

Our study is one of the first to quantitatively assess the effects of daily rainfall within winters on bird condition while accounting for individual capture dates and spatial variation in habitat and moisture. We found support for our fourth hypothesis in the drier winter of 2013, as the standard deviation of daily rainfall during the 90 days prior to capture, interacting with habitat moisture (δ^13^C values), was a better predictor of the size‐corrected mass index than total rainfall and ordinal date. This finding was likely due to the fact that larger precipitation events are more likely to inundate soils with moisture and have lower rates of evapotranspiration compared to smaller precipitation events (Reynolds, Kemp, Ogle, & Fernández, 2004; Thomey et al., 2011). Moreover, rainfall during the 90 days prior to capture predicted size‐corrected mass in 2013 better than rainfall during the 30, 60, or 120 days prior to capture. These results suggest that cumulative amounts of rainfall over a long period are important for birds during drier winters. However, we also found pectoral muscle to be more impacted by total rainfall during the 30 days prior to capture in 2013, which is consistent with a study by Angelier et al. (2011) that showed American Redstarts to be affected relatively quickly by changes in rainfall. In addition, Wunderle et al. (2014) found that Kirtland's Warblers’ condition in late winter was significantly affected by rainfall occurring 30, 60, and 90 days prior to capture, and the effect was most significant during the 30 days prior to capture. In light of our findings and those of other studies, we conclude that birds benefit from both short‐term rainfall pulses (within 30 days) and longer‐term cumulative amounts of rainfall (90 days). In 2014, birds surprisingly had lower muscle scores when rainfall totals in the previous 30 days were higher. Perhaps other unexamined processes were interacting to influence muscle during this wetter year. Theoretically, birds could have been trading‐off muscle maintenance with improvements in plumage quality during pre‐alternate molt in 2014 (Tonra et al., 2014). Regardless of the reason for the 2014 pattern, within‐season rainfall appears to influence bird condition differently during wet versus dry winters.

We found support for our third hypothesis only in the drier winter of 2013, when birds captured in coastal scrub and short buttonwood vegetation declined in both size‐corrected mass and pectoral muscle, but birds in tall coppice vegetation maintained condition. Our study suggests that relatively taller and wetter vegetation in The Bahamas provides better habitat compared to the shorter and drier coastal scrub vegetation. Our results are consistent with studies conducted in different vegetation communities elsewhere in the Caribbean (Marra & Holmes, 2001; Wunderle, 1995), including the study on Prairie Warblers occupying pine forests and desert sites in the Dominican Republic (Latta & Faaborg, 2001). In addition, we found that birds were able to maintain condition in non‐native vegetation, which was wetter than coastal scrub. In Puerto Rico, Baltz (2000) observed that Prairie Warblers were able to maintain body condition over the winter dry season in wooded pastures that were dominated by *Lantana sp*. and non‐native legumes. These findings in Prairie Warblers are consistent with additional studies in other species (Beltrán & Wunderle, 2013; Johnson et al., 2006; Murphy et al., 2001), overall suggesting that non‐native vegetation in the Caribbean may provide migratory birds with moderate‐quality wintering habitat.

We observed habitat segregation in age/sex classes of Prairie Warblers on San Salvador, supporting our first hypothesis; males were more abundant in habitats with taller vegetation, and blood δ^13^C values indicated home ranges in wetter habitats. Females were more likely to be observed in shorter vegetation, and δ^13^C values confirmed their home ranges were in drier habitats. Other studies on Prairie Warblers and other migratory passerines have also found males are more abundant in taller habitats during the winter (Latta & Faaborg, 2001; Murphy et al., 2001; Ornat & Greenberg, 1990; Wunderle, 1995). Male Prairie Warblers forage higher than females on their breeding grounds (Nolan, 1978), and thus, one could postulate that males and females could be innately selecting wintering habitats (Morton, Lynch, Young, & Mehlhop, 1987). However, intraspecific competition and dominance‐related habitat selection are likely structuring habitat occupancy in our study system because habitat segregation was correlated with body condition. Females should not innately select wintering habitats in which their physical condition would be reduced (Marra, 2000; Marra & Holmes, 2001). Our findings that older males occupying taller, wetter habitats had better overwinter body condition compared to the younger and female individuals occupying shorter, drier habitats are consistent with other studies (Latta & Faaborg, 2001, 2002; Mettke‐Hofmann et al., 2015; Studds & Marra, 2005). Importantly, because habitat segregation can lead to pronounced differences in body condition among different age/sex classes, this can subsequently lead to differential carry‐over effects and seasonal interactions (Drake et al., 2013; Marra et al., 1998; Norris et al., 2004).

We note there were a few limitations in our body condition analyses. We had a small sample size of individuals captured in mid‐winter and recaptured in late winter to examine changes in body condition within specific individuals, and we therefore could not test certain hypotheses such as changes in individual‐level body condition among age and sex classes. Furthermore, Smith, Reitsma, and Marra (2011) observed for a different migratory passerine, the Northern Waterthrush, that site‐faithful, territorial birds can maintain body condition better than nonterritorial birds during the winter dry season. Including a covariate of site‐faithfulness would have been useful in our population‐level condition analyses, but unfortunately we could not fully determine which of the captured birds were site‐faithful throughout the winter. Interestingly, our observed findings, such as changes in body condition within specific years and in relation to habitat moisture, were similar from the population‐level (which had site‐faithful and floater birds) and the individual‐level analyses (which had only site‐faithful, recaptured birds). These combined findings indicate that our population‐level analyses were robust despite not accounting for a covariate of site‐faithfulness. Our results suggest that either there was little difference in overwinter changes in body condition of floater versus site‐faithful individuals in our study, or alternatively, if there was a difference, there were too few floater birds to drastically alter the overall population‐level findings.

Our study is one of the first to closely examine effects of both spatial variables (i.e., vegetation type and height) and temporal variables (i.e., year, ordinal date, and rainfall) on δ^13^C values in bird tissue on the wintering grounds. Consistent with other studies and as predicted (hypothesis 2a), δ^13^C in blood appeared to reflect spatial variation in moisture based on vegetation communities (i.e., coastal scrub vs. tall coppice and mangroves; Marra et al., 1998, Bearhop et al., 2004, Smith et al., 2010). We note that classifications of the vegetation were based on the bird capture locations, and in the two plots with heterogeneous habitats, birds may have been using other vegetation types adjacent to their capture locations. Specifically, there were only small, interspersed areas of short, inland coppice in our study sites, such that birds captured in short coppice were resighted primarily in other habitats. As a result, there was a high variance of δ^13^C values for birds captured in short coppice. However, besides short coppice, the vegetation at the capture locations did correlate highly with the vegetation used by resighted birds throughout their home ranges, thus comparing δ^13^C values with the vegetation at the capture locations was generally warranted. Overall, using δ^13^C values as an index of spatial moisture allowed us to assess moisture levels and habitat occupancy beyond the capture location at a home range scale, without radio‐tracking individuals. Unfortunately, we did not examine δ^13^C values in plants or insects, and we therefore assumed that similar spatial patterns between δ^13^C values and habitat moisture would have been observed among all of the corresponding trophic levels, as found in other studies (Flanagan & Johnsen, 1995; Marra et al., 1998; Stewart et al., 1995).

Examining the effects of year, ordinal date, and rainfall on δ^13^C values indicated that there were longer‐term, but not shorter‐term, temporal relationships with δ^13^C values in bird tissues. δ^13^C values in bird blood became enriched (drier) over the winter in the population sample, but δ^13^C values were not significantly different between mid‐ and late winter samples for the recaptured individuals. For recaptured birds, perhaps the average period between each capture and recapture was too short (57 days) to detect the temporal effect of date on δ^13^C, compared to the time frame of captures in the entire population (94 days). Isotope values in blood versus claws were also highly correlated, despite claw samples incorporating a slightly longer isotopic dietary time‐span compared to blood (Lourenço et al., 2015), further suggesting no short‐term temporal effect on δ^13^C values. Among years, 2014 had enriched δ^13^C values (indicating less moisture) compared to 2013, which matches rainfall amounts in September–December, but not in January–March. Finally, we found a significant effect of rainfall 60 to 120 days prior to capture on δ^13^C, but not 30 days prior to capture, again indicating that the δ^13^C values in bird tissue incorporate temporal variation in moisture with a time lag. Similar to our results, Studds and Marra (2005) also observed differences in δ^13^C in American Redstarts sampled among years and found slightly enriched δ^13^C values for individual birds recaptured in the same habitat type in late winter compared to mid‐winter.

δ^13^C values in individual plants can change over time due to seasonal rainfall amounts and drought conditions (Korol et al., 1999; McNulty & Swank, 1995). Given that temporal changes in moisture are first reflected in δ^13^C values in plants during photosynthesis (Farquhar et al., 1989), and then the carbon is passed through herbivorous insects to birds over a period of time (Marra et al., 1998), the time lag that we observed in δ^13^C values in bird tissue as a response to rainfall is biologically intuitive. Nonetheless, further research is needed to confirm that temporal changes in carbon isotopes, due to temporal changes in rainfall, occur as expected along the entire food chain. With respect to solely the δ^13^C values observed in bird tissue, our findings are important to consider when assessing carry‐over effects (e.g., Norris et al., 2004, Drake et al., 2013, Akresh, King, & Marra, 2019), as variation in δ^13^C values among years in samples obtained at arrival to migratory stop‐over sites or breeding grounds may be indicative of temporal rainfall patterns occurring in early to mid‐winter, and not during late winter. Additionally, we note that although using δ^13^C values in our study allowed us to accurately examine the effects of spatial moisture on body condition, the δ^13^C values in bird tissue unfortunately were not indicative of the temporal variation in moisture at the time of capture.

### 4.1 Conservation implications

Rainfall in the Caribbean is expected to decline and also become more variable over the next 50 years (Neelin et al., 2006; Studds & Marra, 2011). Given continued development and deforestation in the tropics and limited resources to conserve habitat (Achard et al., 2014), we recommend that preservation efforts for migratory passerines focus on the least drought‐prone areas (Wunderle et al., 2014), in which The Bahamas are sites where the freshwater table is close to the surface (Sealey, 2006). Conservation areas should include high‐moisture habitats (i.e., mangroves) that have been shown to be of high quality for certain species of wintering Nearctic–Neotropical migrants (Johnson et al., 2006; Smith et al., 2010). Additionally, conservation areas should include relatively wetter sites within other vegetation communities that are often not perceived as “high moisture” (i.e., inland coppice), yet our study found that these areas also provide high‐quality habitat. Conservation of a variety of vegetation communities will not only benefit threatened migrants that are “forest” specialists, such as Swainson's Warblers (*Limnothlypis swainsonii*; Strong & Sherry, 2001) and Kirtland's Warblers (Wunderle et al., 2014), but also resident birds and other wildlife dependent on the preservation of the entire breadth of different vegetation communities (Currie et al., 2005).

Climate change will likely affect birds in complex ways; for instance, some species of migratory birds are adapting to warmer springs in temperate zones by arriving earlier to the breeding grounds (Jonzén et al., 2006), yet lower rainfall in the wintering grounds will make it more difficult for migrants to acquire premigratory reserves and depart earlier on spring migration (Studds & Marra, 2011). Females and younger individuals segregated to poorer‐quality, drier habitats may be especially prone to predicted declines in rainfall (Marra & Holmes, 2001), which may subsequently affect processes on the breeding grounds (Drake et al., 2013; Norris et al., 2004). Our study shows that Prairie Warbler populations could be limited by habitat quality during the wintering season, although more studies of effects on fitness (e.g., survival) are needed. Continued research on full annual cycles of migratory passerines is important to fully understand limiting factors of populations and provide best management tools for declining and threatened species (Marra, Cohen, et al., 2015a).

## CONFLICT OF INTEREST

None declared.

## AUTHOR CONTRIBUTIONS

All authors conceived the research idea, contributed to designing methodology, and coordinated data collection and/or laboratory work. MEA conducted statistical analyses and wrote the manuscript. All authors interpreted the results, revised the manuscript, and gave final approval for publication.

## Data Availability

The data used in this study are archived in the Dryad data repository: https://doi.org/10.5061/dryad.4435q76.

## APPENDIX A

**Table A1:** Candidate model set examining rainfall variables and date on Prairie Warbler size‐corrected mass

Model No.	Fixed‐effect Variables	*K*
1	Time	4
2–14	Time+(Rainfall Variable or Date)	5
15–27	Time+(Rainfall Variable or Date)*δ^13^C	7
28–40	Time+(Rainfall Variable or Date)*VegHtatNet	7
41–53	Time+(Rainfall Variable or Date)*VegTypeatNet	15

Note

We examined a similar model set for pectoral muscle score as the response variable, but time (time after sunrise) was not included in pectoral muscle models and thus those models had one less parameter. All models included a random effect of individual. The rainfall variable in a given model was either total, *SD*, or CV of daily rainfall during the 30, 60, 90, or 120 days prior to capture. VegHtatNet = vegetation canopy height at the capture net, VegTypeatNet = vegetation type at the capture net, *K* = number of parameters.

**Table A2:** Age and sex of Prairie Warblers captured among vegetation types in San Salvador Island, between 2012 and 2015. Presented are within‐vegetation‐type percentages, with total numbers (*n*) in parentheses

	Short Buttonwood	Coastal Scrub	Mangrove and Sabal Palm	Non‐native	Short Coppice	Tall Coppice
Juvenile Female	47 (23)	42 (58)	13 (2)	41 (26)	60 (6)	24 (12)
Adult Female	16 (8)	24 (33)	0 (0)	24 (15)	20 (2)	12 (6)
Juvenile Male	24 (12)	18 (25)	73 (11)	21 (13)	0 (0)	45 (22)
Adult Male	12 (6)	16 (22)	13 (2)	14 (9)	20 (2)	18 (9)
Total Male	36 (18)	34 (47)	86 (13)	35 (22)	20 (2)	63 (31)
Total Adult	28 (14)	40 (55)	13 (2)	38 (24)	40 (4)	30 (15)
